# Phloretamide Protects against Diabetic Kidney Damage and Dysfunction in Diabetic Rats by Attenuating Hyperglycemia and Hyperlipidemia, Suppressing NF-κβ, and Upregulating Nrf2

**DOI:** 10.3390/pharmaceutics16040505

**Published:** 2024-04-07

**Authors:** Rasha Al-Hussan, Nawal A. Albadr, Ghedeir M. Alshammari, Soheir A. Almasri, Farah Fayez Alfayez, Mohammed Abdo Yahya

**Affiliations:** 1 Department of Food Science and Nutrition, College of Food and Agricultural Sciences, King Saud University, Riyadh 11451, Saudi Arabia; 437203574@student.ksu.edu.sa (R.A.-H.); nalbader@ksu.edu.sa (N.A.A.); aghedeir@ksu.edu.sa (G.M.A.); saalmasry@ksu.edu.sa (S.A.A.); 2 Department of Medicine and Surgery, College of Medicine, King Saud University, Riyadh 11451, Saudi Arabia; farahfayezm@gmail.com

**Keywords:** phloretamide, kidney, diabetes, oxidative stress, Nrf2

## Abstract

Potent hypoglycemic and antioxidant effects were recently reported for the apple-derived phenolic compound phloretamide (PLTM). The renoprotective effects of this compound are yet to be shown. This study aimed to examine the potential of PLTM to prevent diabetic nephropathy in streptozotocin-induced diabetic rats and to examine the possible mechanisms of protection. Non-diabetic and STZ-diabetic male rats were treated orally by gavage with either the vehicle or with PTLM (200 mg/kg; twice/week) for 12 weeks. PTLM significantly increased urine volume and prevented glomerular and tubular damage and vacuolization in STZ-diabetic rats. It also increased creatinine excretion and reduced urinary albumin levels and the renal levels of kidney injury molecule-1 (KIM-1), 8-hydroxy-2′-deoxyguanosine (8-OHdG), neutrophil gelatinase-associated lipocalin (NGAL), and nephrin in the diabetic rats. PTLM also prevented an increase in the nuclear levels of NF-κβ, as well as the total levels of tumor necrosis factor-alpha (TNF-α), interleukin 6 (IL-6), caspase-3, and Bax in the kidneys of diabetic rats. These effects were associated with reduced serum levels of triglycerides, cholesterol, and low-density lipoprotein cholesterol. In both the control and diabetic rats, PTLM significantly reduced fasting plasma glucose and enhanced the renal mRNA and cytoplasmic levels of Nrf2, as well as the levels of Bcl2, superoxide dismutase (SOD), and glutathione (GSH). However, PTLM failed to alter the cytoplasmic levels of keap1 in diabetic rats. In conclusion, PTLM prevents renal damage and dysfunction in STZ-diabetic rats through its hypoglycemic and hypolipidemic activities, as well as through its antioxidant potential, which is mediated by activating the Nrf2/antioxidant axis.

## 1. Introduction

Diabetic nephropathy (DN) is a well-known complication that develops in 30% and 40% of patients with type 1 diabetes mellitus (T1DM) and type 2 diabetes mellitus (T2DM), respectively [1,2]. DN is associated with global health and socioeconomic burdens and all-cause mortality and is a leading cause of the development of chronic kidney disease (CKD), cardiovascular disorders (CVDs), and end-stage renal disorders (ESRDs) [3]. Generally, DN is characterized by severe damage to small blood vessels (angiopathy), hypertension, albuminuria, reduced glomerular filtration rate (GFR), thickening of the glomerular basement membrane, deposition of extracellular matrix proteins, and alterations in renal hemodynamics [2,4]. Currently, no therapy is available to treat DN [5]. Therefore, a better understanding of the molecular basis of the pathogenesis of the disease is a priority in identifying a suitable and safe treatment.

On the other hand, the pathogenesis of DN is well established and includes three major mechanisms, namely, alterations in hemodynamic, metabolic, and inflammatory pathways [2]. Yet, the use of antidiabetic medications to control blood glucose or blood pressure parameters fails to improve the high death rate due to the renal and cardiovascular events associated with CKD [1]. This could be explained by the complexity of this disorder and suggests focusing more on intra-renal mechanisms that usually develop in the presence of hyperglycemia and tissue hypoxia. Within this context, it was shown that DN is an inflammatory disorder that involves various levels of oxidative stress, inflammation, fibrosis, and necrosis, which result in renal glomerular and tubular damage [2]. However, accumulating data indicate that oxidative stress and inflammation are the two upstream mechanisms initiating the incidence of DN under hyperglycemia and hyperlipidemia [6,7,8]. Yet, both mechanisms crosstalk and act in a vicious cycle to potentiate each other. Interestingly, several lines of evidence suggest that the role of oxidative stress is the most prominent in mediating DN, being a central mechanism that induces inflammation, fibrosis, necrosis, and apoptosis [9,10,11]. Indeed, in the early stages of diabetic kidney damage, hyperglycemia induces massive amounts of reactive oxygen species (ROS) through mitochondria, polyol, hexosamine, and advanced glycation end product (AGE) pathways and by activating several targets such as protein kinase C and nicotinamide adenine dinucleotide phosphate (NADPH) oxidase (NOX) [2,12]. In turn, ROS not only induces renal tissue lipid peroxidation but also exaggerates renal damage by overwhelming the renal antioxidant systems, inducing podocyte injury, and disturbing the glomerular filtrating membrane [10]. In addition, hyperglycemia alone or through inducing ROS triggers renal inflammation and the recruitment of inflammatory cells, as well as interstitial fibrosis by increasing the transcription of several inflammatory cytokines and adhesive molecules in several renal cells, including podocytes, mesangial cells, macrophages, vascular endothelial cells, and renal tubule cells by regulating several transcription factors and regulatory molecules [10,11]. These include nuclear factor kappa beta (NF-κB), transforming growth factor beta1 (TGF-β1), interleukin 6 (IL-6), tumor necrosis factor-alpha (TNF-α), NRLP3 inflammasome, and intracellular cell adhesive molecule (ICAM) [8,11]. However, treatment with antioxidants alleviated DN and prevented the progression to CKD and ESRD in animal models by suppressing inflammation, podocyte injury, tubular interstitial fibrosis, and apoptosis, suggesting that oxidative stress is the upstream mechanism underlying the pathogenesis of DN. 

Nuclear factor erythroid 2-related factor 2 (Nrf2) and NF-κβ are the two major transcription factors in the cells that stimulate antioxidants and suppress inflammation, respectively [13]. Nrf2 and NF-κB are resident cytoplasmic proteins that are inhibited by binding to other cytoplasmic proteins [13]. Upon oxidative stress, both factors are activated and transported to the nucleus to initiate the transcription of several target genes [14,15]. Both Nrf2 and NF-κB are involved in the pathogenesis of DN, where they can negatively affect their activation. On the one hand, NF-κB is abnormally hyperactivated in the kidneys of diabetic animals [16]. Hyperglycemia, ROS, AGEs, cytokines, and ANG II can rapidly activate NF-κβ in the majority of kidney-resident cells [17]. This protein can further stimulate renal cell inflammation, fibrosis, and oxidative stress by accumulating macrophages, increasing the expression of inflammatory cytokines and adhesive markers, increasing the generation of ROS, and suppressing antioxidant expression by antagonizing Nrf2 signaling [18,19,20]. On the other hand, Nrf2 is a protective signal against diabetic nephropathy by promoting the expression of enzymatic and nonenzymatic antioxidants such as glutathione (GSH), heme oxygenase 1 (HO-1), catalase, and superoxide dismutase (SOD). Nrf2 signaling is severely impeded in DN and is associated with oxidative stress, inflammation, apoptosis, and the over-activation of NF-κβ [21,22]. However, attenuating the increase in the activity of NF-κβ or the activation of Nrf2 significantly improved renal function and reduced renal damage in diabetic animal models [18,21,23].

Unfortunately, diabetic medications are associated with adverse effects [24]. Therefore, there is an urgent need to find suitable and safe therapies to slow the progression and improve the diagnosis and treatment of DN. Currently, there is great interest in discovering therapeutic agents from plant ingredients, given their potent potential to lower blood glucose and their antioxidant and inflammatory properties. To date, tens of plant polyphenols have been reported to treat DN by lowering fasting hyperglycemia and/or antagonizing renal oxidative stress and inflammation. Examples are resveratrol, allicin, quercetin, and ursolic acid [19]. Phloretin (C_15_H_14_O_5_), a major flavonoid in apples, is another example of an antidiabetic drug that can effectively reduce hyperglycemia and fight oxidative stress in other chronic disorders. The free radical scavenging activity of phloretin was observed in vitro using the 2,2-Diphenyl-1-picrylhydrazyl (DPPH) antioxidant assay [25]. In addition, several in vitro and in vivo studies have documented that phloretin can inhibit inflammation, the synthesis of inflammatory cytokines, and oxidative apoptotic damage in a variety of disorders through its potential to suppress NF-κB and activate Nrf2 (reviewed in [25]). Phloretamide (PHLTM) is a 3-(p-Hydroxyphenyl) propionic acid, another phenolic compound that is derived from the metabolism of phloretin. The discovery of PHLTM occurred in the mid-1980s. Rybicka showed that PHLTM is abundantly found in the fruits and root exudates of apple trees. The pharmacological properties of PHLTM have not been investigated. However, recent updates from our laboratories and others suggest that PHLTM has potent antioxidant and anti-inflammatory effects. Indeed, Krajka et al. [26] demonstrated an exceptional potential of PHLTM to activate Nrf2 in cultured hepatocytes. Based on this evidence, we also showed that treatment with 100 or 200 mg/kg alleviated liver damage and steatosis in STZ-induced T1DM in rats. The mechanisms of protection were the lowering of fasting hyperglycemia, the attenuation of hepatic gluconeogenesis and dyslipidemia, the activation of Nrf2, the upregulation of antioxidants, and the suppression of NF-κβ and inflammatory cytokine production [27].

However, the nephroprotective effect of PHLTM has not been tested in an animal model of kidney injury. Based on our previous observations in diabetic rats and on the potential exhibited by PHLTM, in this study, we assumed that co-treatment with PHLTM could also prevent the development of DN in STZ-induced T1DM in rats. In addition, we tested the possible mechanisms of action, including the effect on markers of oxidative stress and inflammation and on the activities of Nrf2 and NF-κβ.

## 2. Materials and Methods

### 2.1. Animals

For the study, we used adult male Wistar rats (150–170 g, 9 weeks old). The animals were supplied by the Experimental Animal Care Center at King Saud University, Riyadh, Saudi Arabia. All rats were housed as 4 males in one cage in separate rooms at 22 ± 5 °C. The day/night cycle was 12 h for each. Water was delivered to the animals, and all rats had free access. During the experiment, the control and diabetic rats were fed a normal growth diet of 105 fat (cat # D12450B, Research Diets, New Brunswick, NJ, USA). All experimental protocols of this study were approved by the Animal Use and Care Ethical Committee at King Saud University (IRB number: KSU-SE-22-58).

### 2.2. Induction of STZ-Mediated T1DM

We previously showed that DN develops in STZ rats by 10–12 weeks following the single intraperitoneal injection of STZ (65 mg/kg) [28]. This was also followed in this study to induce T1DM in experimental rats. In accordance, STZ (Cat. No. S0130, Sigma Aldrich, St. Louis, MO, USA) was first dissolved in a slightly acidic citrate buffer at a final concentration of 65 mg/kg and i.p. injected at this volume to selected rats. Using this model, we previously showed that such a dose of STZ administered over 3 days can damage the majority of pancreatic beta cells and raise fasting blood glucose levels to higher than 300 mg/kg. Hence, T1DM was confirmed in the rats of this study by measuring the fasting hyperglycemia levels (>300 mg/kg) in their blood samples, which were obtained from their tails and measured using a glucometer. These diabetic rats were further included in the experimental design as shown below.

### 2.3. Experimental Design

PHLTM was provided by Yangzhou Chemical Co., Ltd., Jiangsu, China; PHLTM was reported in our previous work [27]. PHLTM was freshly prepared in 0.5% carboxymethylcellulose (CMC) (#5678, Sigma Aldrich, MO, USA) to a final concentration (200 mg/kg). PHLTM was administered orally to all rats using a stainless steel gavage needle in 2 doses/week (each 200 mg/kg; 0.5 mL). The control and non-diabetic rats were divided into 4 groups of 8 rats: (1) control group: control rats that were orally treated with normal saline as a vehicle (0.5 mL; twice a week); (2) T1DM group: diabetic rats that were orally treated with normal saline (0.5 mL; twice a week); (3) control + PHLTM group: control rats that were orally treated with 0.5 mL PHLTM (200 mg/kg; twice a week); and (4) T1DM + PHLTM group: diabetic rats that were treated with 0.5 mL PHLTM (200 mg/kg; twice a week). The treatments ended after 12 weeks.

### 2.4. Dose Selection

The dose of PHLTM (200 mg/kg) was selected based on our previous findings, which showed hypoglycemic, hypolipidemic, hepatic antioxidant, and anti-inflammatory effects in STZ-diabetic rat models [27]. In addition, the hepatic protective effect of this dose afforded hepatic protection by activating Nrf2 and suppressing NF-κβ.

### 2.5. Urine and Blood Sample Collection and Analyses

One day after the last treatment, 24 h urine samples were collected from all groups of animals using special Tecniplast metabolic cages for rats provided with water and food. Then, all rats were fasted for another 12 h and were anesthetized with ketamine hydrochloride solution (80 mg/kg). Blood samples were collected from all rats via cardiac puncture, and fractions were placed into either gel-containing or EDTA-containing tubes. These tubes were allowed to be set at room temperature for 30 min and then centrifuged at 500× *g* for 15 min. Supernatants representing serum and plasma samples, as well as all collected filtered urine samples, were directly collected and maintained at −20 °C.

### 2.6. Biochemical Analysis of the Plasma, Serum, and Urine

The levels of glucose and inulin in the plasma samples of all groups were evaluated using special assay and ELISA kits for rats (# 10009582 and #589501, Cayman Chemicals, Ann Arbor, MI, USA). Serum levels of total cholesterol, triglycerides, high-density lipoprotein cholesterol (HDL-c), free fatty acids (FFAs), and low-density lipoprotein cholesterol (LDL-c) were measured using assay kits provided by AFG Scientific, Northbrook, IL, USA (# EK720559, # EK720636, # EK720660, # EK721336, # EK720763). Urinary and serum levels of creatinine and albumin were measured using ELISA kits (# EK720897 AFG Scientific, Northbrook, IL, USA and # 80662, Crystal Chem, Elk Grove Village, IL, USA). Urinary levels of kidney injury molecule-1 (KIM-1), 8-hydroxy-2′-deoxyguanosine (8-OHdG), neutrophil gelatinase-associated lipocalin (NGAL), and nephrin were evaluated using ELISA (# EK720751, AFG Scientific, Northbrook, IL, USA; EK720424, AFG Scientific, Northbrook, IL, USA; # 80687 Crystal Chem, Elk Grove Village, IL, USA, CSB-E13957r, CusaBio, Houston, TX, USA, respectively). All kits used for these measurements were rat-specific. All analyses were performed in duplicate for *n* = 8 samples/group as per the kits’ instructions. 

### 2.7. Kidney Collection and Processing

After blood collection, all animals were euthanized via cervical dislocation. The right and left kidneys were dissected out onto ice and further cut into smaller pieces. Some kidney parts from all groups of rats were fixed in 10% buffered formalin and sent to the pathology laboratory at the College of Science for further histological processing. The remaining kidney parts were quickly placed in liquid nitrogen and kept at −80 °C until further processing. Later, some parts of these frozen kidneys were homogenized in neutral phosphate-buffered saline and centrifuged (10,000× *g*/10 min/4 °C) to collect tissue homogenates (supernatant). Other parts were used to extract the nuclear and cytoplasmic fraction using a commercial nuclear/cytoplasmic separation kit (# 40010, Active Motif, Carlsbad, CA, USA).

### 2.8. Biochemical Analysis of the Renal Homogenates

The levels of IL-6, TNF-α, GSH, SOD, and HO-1 in the kidney homogenates were measured using ELISA kits (# EK720267 and # EK720127, (# EK720816, # EK720188, # EK720889, and # EK720658, AFG Scientific, Northbrook, IL, USA). The levels of the advanced glycation end products (AGEs) were evaluated using ELISA (# CSB-E09413r, CusaBio, Houston, TX, USA). The levels of Bcl2-associated X protein (Bax) in the renal homogenates were measured using an ELISA kit (BioVision, E4513, Milpitas, CA, USA). ELISA kits were used to measure the levels of B-cell lymphoma 2 protein (Bcl2) and caspase-3 (LS-F11016 and # LS-F4135, LS Bio, Lynnwood, WA, USA). All ELISA kits used were specific to rats, and all measurements were conducted in duplicate and for *n* = 8 samples/group. All protocols were conducted as described by each kit’s manufacturer.

### 2.9. Biochemical Analysis of the Cytoplasmic and Nuclear Extracts

The cytoplasmic and nuclear levels of Nrf2 and NF-κβ in the pre-prepared cytoplasmic and nuclear fractions were determined using special rat ELISA kits (# 50296 and # 31102, Active Motif, Carlsbad, CA, USA). All procedures were carried out for 8 samples/group as per the provider’s instructions.

### 2.10. Real-Time PCR (qPCR)

The levels of transcription of Nrf2, keap1, NF-κβ, and β-actin (control) were determined using qPCR primers for these parts, as per our previous study [28]. Briefly, the total RNA was isolated from each frozen kidney sample (30 mg) using the TRIZOL reagent. The first strand of DNA was prepared using a commercial kit (# ab 286905). cDNA amplification was conducted using a real-time PCR machine (model number CFX96, BioRad, Hercules, CA, USA) with the help of the Ssofast Evergreen Supermix kit (#1725201, BioRad) as per the instructions. The levels of expression of Nrf2, keap1, and REDD1 were recorded as a percentage of the control (β-actin). Analysis was carried out for 8 samples/group.

### 2.11. Statistical Analysis

GraphPad Prism Analysis Software (version 8, Bengaluru, Karnataka, India) was used for the analysis of the data. First, we tested the normality of all collected data using the Kolmogorov–Smirnov test. The comparison between the control and HFD group with all treatments was conducted using two-way ANOVA, which was followed by Tukey’s post hoc test. The data were considered significantly different if *p* < 0.05 and were presented as means ± standard deviation (SD). 

## 3. Results

### 3.1. Effect of PHLTM on Body Weight and Metabolic Parameters

No death was observed in all groups of rats in this study. The final body weights were not significantly different between the control and control + PHLTM rats but were significantly reduced in the STZ-T1DM rats compared to both groups (Table 1). The final body weights were significantly increased in the STZ-T1DM + PHLTM rats compared to the STZ-T1DM rats (Table 1). Only fasting plasma glucose was significantly lower in the control + PHLTM rats compared to the control rats. However, the fasting plasma or serum levels of insulin, TGs, CHOL, and LDL-c were not significantly different between the control and control + PHLTM rats (Table 1). Fasting blood glucose and serum TGS, CHOL, and LDL-c were significantly higher, but fasting plasma glucose and serum HDL-c were significantly lower in the STZ-T1DM rats compared to the control rats (Table 1). The serum insulin levels were not significantly different between the STZ-T1DM rats and STZ-T1DM + PHLTM rats (Table 1). On the other hand, the STZ-T1DM + PHLTM rats showed significantly lower levels of fasting glucose, TGs, CHOL, and LDL-c and higher levels of HDL-c compared to the STZ-T1DM rats (Table 1). None of these parameters reached their basal levels in the control groups (Table 1).

### 3.2. Effect of PHLTM on Markers of Kidney Function

None of the measured kidney function markers were statistically different between the control and control + PHLTM rats (Table 2). There was a significant increase in the serum levels of creatinine that was parallel with a significant reduction in serum albumin levels in the STZ-T1DM rats compared to the control and the control + PHLTM-treated rats (Table 2). Also, the urine volume and urinary creatinine levels were significantly reduced, but urinary levels of albumin, 8-OHdG, KIM-1, nephrin, and NGAL were significantly increased in the STZ-T1DM compared to the control or control + OHLTM rats (Table 2). The serum levels of albumin and urinary levels of creatinine were significantly increased, but serum creatinine levels and urinary levels of 8-OHdG, KIM-1, nephrin, and NGAL were significantly decreased in the STZ-T1DM + PHLTM rats compared to the STZ-T1DM rats (Table 2).

### 3.3. Effect of PHLTM on Renal Markers of Oxidative Stress and Inflammation

With no significant variation in the levels of AGEs, TNF-α, IL-6, Bax, and caspase-3, the levels of MDA were significantly reduced, and levels of GSH, SOD, HO-1, and BCl2 were significantly higher in the kidneys of the control + PHLTM rats compared to the control rats (Table 3 and Table 4). The levels of MDA, AGEs, TNF-α, IL-6, Bax, and caspase-3 were significantly higher, but the levels of SOD, HO-1, GSH, and Bcl2 were significantly lower in the kidneys of the T1DM-STZ rats compared to the control and control + PHLTM rats (Table 3 and Table 4). The kidneys of the STZ-T1DM rats showed a significant reduction in their renal levels of MDA, AGEs, TNF-α, IL-6, caspase-3, and Bax, concomitant with an increase in the levels of SOD, HO-1, GSH, and Bcl2, compared to the T1DM-STZ rats (Table 3 and Table 4).

### 3.4. Changes in the Expression Levels of Nrf2 and NF-κβ

**Figure 1:**
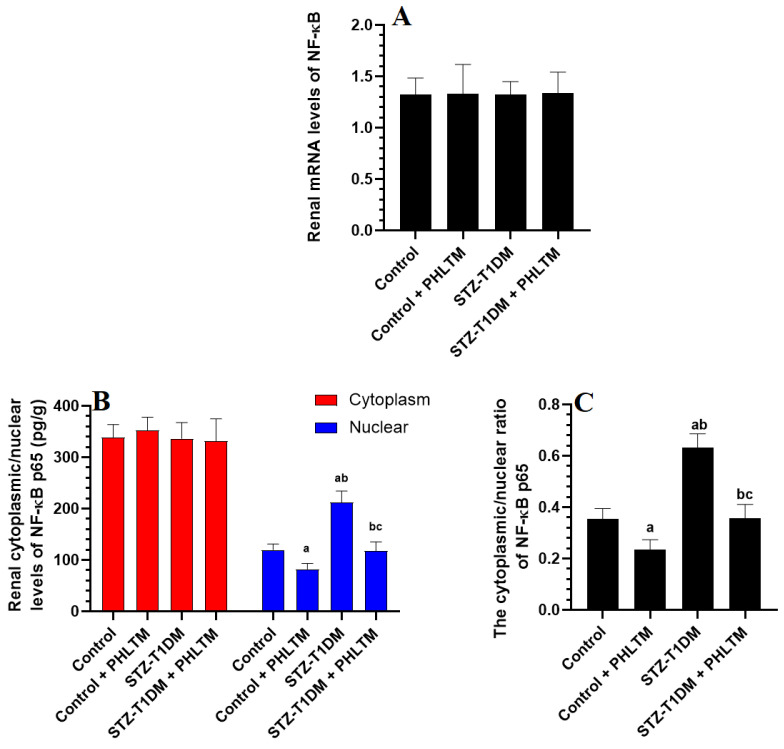
mRNA expression of NF-κβ (**A**), renal cytoplasmic and nuclear levels of NF-κB p65 (**B**), and the ratio of cytoplasmic/nuclear levels of NF-κβ p65 (**C**) in the kidneys of all experimental groups. Analysis was conducted using two-way ANOVA followed by Tukey’s test. ^a^ Significantly different from control (non-diabetic) rats (control). ^b^ Significantly different from control (non-diabetic) + PHLTM rats. ^c^ Significantly different from STZ-T1DM-treated rats.

**Figure 2:**
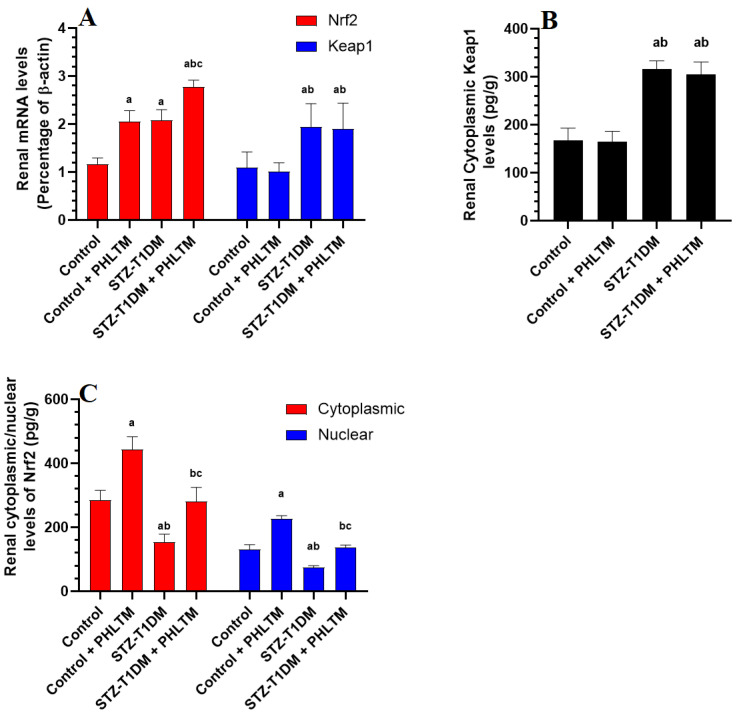
mRNA levels of Nrf2 and Keap1 (**A**), Cytoplasmic levels of keap1 (**B**), and cytoplasmic and nuclear levels of Nrf2 (**C**) in the kidneys of all experimental groups. Analysis was conducted using two-way ANOVA followed by Tukey’s test. ^a^ Significantly different from control (non-diabetic) rats (control). ^b^ Significantly different from the control (non-diabetic) + PHLTM rats. ^c^ Significantly different from STZ-T1DM-treated rats.

The renal mRNA and total cytoplasmic levels of NF-κβ were not significantly different between all groups of rats (Figure 1A). The nuclear levels of NF-κβ, as well as the cytoplasmic/nuclear ratio of NF-κβ, were significantly reduced in the control + PHLTM rats but increased in the kidneys of the STZ-T1DM compared to control rats (Figure 1B). The nuclear levels of NF-κβ and the cytoplasmic/nuclear ratio of NF-κβ were significantly reduced in the kidneys of the STZ-T1DM compared to STZ-T1DM rats (Figure 1C). With no alterations in keap1 mRNA levels, the mRNA levels and cytoplasmic protein levels of Nrf2 and nuclear/cytoplasmic ratio of Nrf2 significantly increased in the kidneys of the control + PHLTM rats compared to the control rats (Figure 2A–C). The kidneys of the STZ-T1DM rats showed significantly higher mRNA levels of Nrf2 and keap1, higher levels of keap1, and lower nuclear/cytoplasmic levels of Nrf2 compared to the control and control + PHLTM rats (Figure 2A–C). This was reversed in the kidneys of the STZ-T1DM + PHLTM rats (Figure 2A–C).

### 3.5. Effect of PHLTM on Kidney Morphology

**Figure 3:**
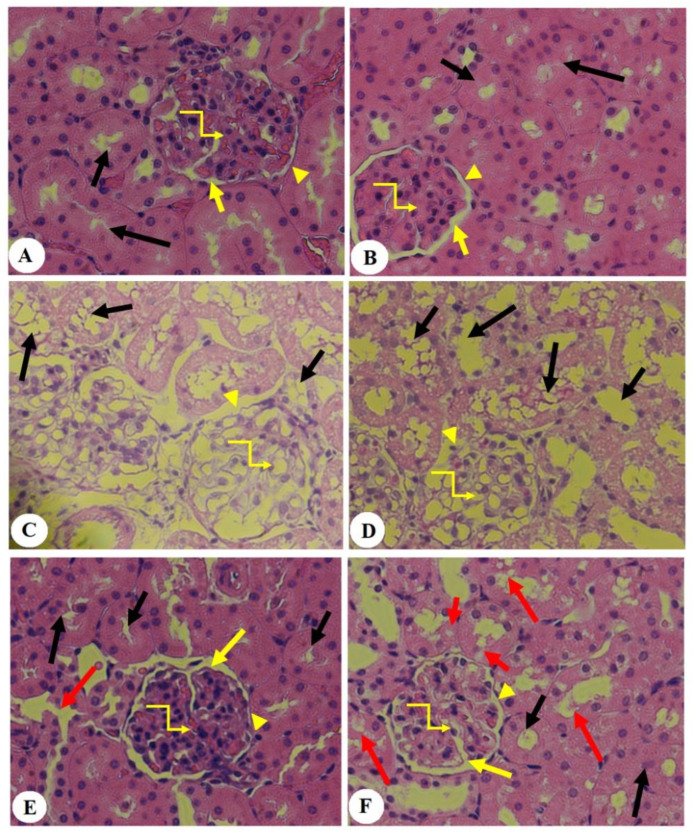
Renal morphology in all groups of rats. (**A**,**B**): Samples obtained from control and control + PHLTM rats showed a normal glomerular structure with intact Bowman’s capsule (yellow arrowhead), normal Bowman’s space (yellow arrow), glomerulus (curved yellow arrow), proximal convoluted tubules (PCTs) (short black arrow), and distal convoluted tubules (DCTs) (long black arrow). (**C**,**D**): Samples obtained from kidneys of STZ-T1DM rats showed a reduced glomerular capillary mass (curved yellow arrow), damaged Bowman’s membrane (yellow arrowhead), and increased vacuolization in the majority of the PCTs (short black arrow) and DCTs (long black arrow). (**E**,**F**): Samples obtained from STZ-T1DM + PHLTM rats showed an almost normal glomerular capillary mass (curved yellow arrow), intact Bowman’s membrane, and increased numbers of PCTs and DCTs with normal epithelium (short and long black arrows, respectively). However, some vacuolization is still seen in the PCTs and DCTs (short and long red arrows, respectively).

The kidneys of the control and control + PHLTM rats showed a normal glomerular structure with intact Bowman’s capsule, intact Bowman’s space, glomerulus, and structures of both the proximal and distal convoluted tubules (PCTs and DCTs) (Figure 3A,B). The kidneys of the STZ-T1DM rats showed an obvious reduction in the glomerular capillary mass, damaged Bowman’s membrane, and increased vacuolization in the majority of the PCTs and DCTs (Figure 3C,D). However, the kidneys of the STZ-T1DM + PHLTM rats showed an almost normal glomerular capillary mass, intact Bowman’s membrane, and an increased number of PCTs and DCTs with a normal epithelium. However, some vacuolization was still seen in the PCTs and DCTs in the kidneys of these rats (Figure 3E,F).

## 4. Discussion and Conclusions

The salient findings of this study demonstrate a significant protective effect of PHLTM against STZ-induced DN in rats, as a model of insulin deficiency and T1DM. In addition, the findings reveal that the mechanism of action involves different arms, including hypoglycemic, hypolipidemic, antioxidant, and anti-inflammatory effects. However, the antioxidant and anti-inflammatory effects afforded by PHLTM are, at minimum, mediated by the activation of the keap1/Nrf2/antioxidant axis and the suppression of NF-κB. 

In mice and rats, STZ-induced insulin deficiency is associated with glomerular and tubular dilation, atrophy, and vacuolation [29,30]. In rats, several authors have induced DN using a high single dose of STZ (55–65 mg/kg) for at least 8 weeks [28,29,31,32]. In addition, the evaluation of acute and chronic kidney disease is based on pathological and clinical findings, including a reduction in the GFR > 50%, oliguria/anuria, the existence of protein/albuminuria, and reduced Cr clearance, as well as depicting some markers of glomerular injury or damage in the urine [29,33]. KIM-1 is a major glycoprotein that is synthesized in low quantities in the proximal tubules of normal kidneys and released in large quantities in the urine upon ischemia or nephrotoxic injury, as well as in response to renal metabolic disturbance [34,35]. Indeed, KIM-1 is the most sensitive marker of tubular injury [35,36]. On the other hand, NGAL, also known as renal troponin, is normally produced by the loop of Henle and neutrophils at low, barely detectable levels. However, urinary levels of NGAL are massively increased in response to tubular injury and inflammation [36]. Nephrin is another transmembrane protein that is normally released from podocytes, and its higher urinary levels indicate early glomerular damage and podocytopathies [37]. The urinary levels of KIM-1, nephrin, and NGAL are significantly increased and used as renal damage markers of DN in both T1DM and T2DM [38,39,40,41]. 

Similar increments in the urinary levels of KIM-1, NGAL, and nephrin, which were concomitant with the obvious histological damage of the glomeruli and renal tubules, were also observed in the STZ-induced diabetic rats of this study. In addition, these diabetic rats showed typical signs of chronic kidney disease, characterized by oliguria, microalbuminuria (10-fold increase), and reduced Cr excretion [29]. On the other hand, the reversal of all of these markers by PHLTM was our first line of evidence for the renoprotective effect of these molecules under such diabetic conditions. The nephroprotective effect of PHLTM has not yet been examined in an animal model of acute or chronic kidney disease. Yet, PHLTM is metabolically derived from phloretin. The nephroprotective potential of phloretin has been widely documented in several other animal studies of chronic renal damage induced by hyperuricemia, cisplatin, diabetes, ischemia, and obesity [42,43,44,45]. 

On the other hand, hypoinsulinemia, hyperglycemia, and dyslipidemia are related conditions that increase the risk of DN and other complications due to their ability to generate massive quantities of ROS and alter cellular metabolism [46,47,48]. Typical features of diabetes-associated dyslipidemia are high circulatory levels of cholesterol, FFAs, TGs, and LDL-c, with low levels of HDL-c [46]. However, a lack of insulin stimulates hepatic lipogenesis and promotes dyslipidemia by decreasing the activity of lipoprotein lipase activity, suppressing lipogenesis in the muscles and adipose tissues, and increasing the influx of FFAs from these peripheral tissues [46]. These events result in muscle wasting and loss of fat pads, which induce a rapid and significant reduction in total body mass in patients with T1DM [49]. In addition, hyperglycemia can induce glomerular and tubular oxidative and inflammatory damage by synthesizing AGEs and promoting protein glycation [50].

In this study, we show that the renal protective effect of PHLTM primarily involves its potential to attenuate fasting hypoglycemia and hyperlipidemia in diabetic rodents. In addition, PHLTM is also able to reduce fasting plasma glucose levels, not only under diabetic conditions but also under basal control circumstances, which confirms its hypoglycemic effect. This supports our previous study in which we showed a similar hypoglycemic effect, which was associated with suppressing hepatic gluconeogenesis key enzymes (glucose-6 phosphatase (G-6Pase) and fructose bisphosphate 1 (FBP-1)) and activating the glycolytic enzyme, hexokinase [27]. On the other hand, PHLTM failed to modulate the insulin levels in the control rats but significantly increased the circulatory levels of insulin in the diabetic ones. Therefore, such a hypoglycemic effect of PHLTM in diabetic rats could be partially attributed to its ability to stimulate insulin secretion from the remaining pancreatic B-cells post-STZ administration [27]. We also show the exceptional dose-dependent ability of PHLTM to regenerate pancreatic beta cells in STZ-diabetic rats [27]. This could explain why only the STZ + PHLTM-treated rats exhibited a significant increase in their body weight and lower levels of FFAs, TGS, CHOL, and LDL-c, effects that are attributed to higher insulin peripheral effects, which stimulate lipid and glycogen synthesis in the adipose tissue and muscles. It could also be possible that PTHLM improved peripheral insulin sensitivity, which was not examined here. 

On the other hand, diabetic nephropathy is described as an inflammatory disorder that develops due to the over-synthesis of inflammatory cytokines and the activation of diverse inflammatory signaling pathways [51,52,53]. These include transcription factors, proinflammatory cytokines (e.g., IL-6, IL-1, IL-8, and TNF-α), chemokines, and adhesive molecules (e.g., ICAM). NF-κβ is the major transcription factor responsible for promoting inflammation by stimulating the synthesis of different inflammatory cytokines and adhesive factors [52]. TNF-α and IL-6 are two major proinflammatory cytokines that are upregulated in the diabetic kidney and are directly linked to the onset of DN [53]. The chronic renal activation of NF-κβ and increased urinary and blood levels of TNF-α and IL-6 were observed in patients and animal models with T1DM [53]. Suppressing TNF-α improved renal function and reduced the markers of glomerular and tubular damage [54]. Likewise, higher levels of IL-6 were correlated with albuminuria and increased epithelial cell permeability, renal hypertrophy, podocyte and tubular injury, and intestinal fibrosis [55]. Nonetheless, renal inflammation in diabetic kidneys is induced mainly by hyperglycemia or independently by other factors such as ROS and FFA [10]. Indeed, hyperglycemia is an independent factor that can directly stimulate IL-12 production in the macrophages, which, in turn, exaggerates inflammation through the production of IFN-γ from the CD4 cells [56]. In addition, a hyperglycemia-mediated increase in renal AGE levels stimulates inflammation via the direct activation of NF-κβ and IFN-γ [20]. In addition, infiltrated FFAs and macrophages/hyperglycemia-derived ROS are other activators of NF-κB and the NRLP3 inflammasome [20]. 

Supporting these data, inflammation was also evidenced in the kidneys of the STZ-diabetic animals of this study by the significant increase in the nuclear activity of NF-κB and higher levels of both TNF-α and IL-6. This could be, as suggested above, due to the synergistic effects of hyperglycemia, ROS, FFAs, and AGEs. However, PHLTM prevented the activation of NF-κB and reduced the renal levels of both inflammatory cytokines. This anti-inflammatory effect of PHLTM could be explained by its ability to reduce the risk factors that pathologically activate NF-κβ, including fasting hyperglycemia, AGEs, ROS, and FFAs [20]. This can be supported by the observation that PHLTM failed to alter the activity of NF-κB and levels of TNF-α and IL-6 in the kidneys of the control rats treated with this drug. Therefore, we could speculate that the observed anti-inflammatory effect of PHLTM is secondary to its hypoglycemic, hypolipidemic, and antioxidant effects. Similar to these data, we also showed the anti-inflammatory effect of PHLTM in the livers of STZ-diabetic animals, which was also attributed to similar mechanisms [27]. This effect of PHLTM could be reflected by the well-reported anti-inflammatory effect of phloretin. Indeed, PHLTM reduced the expressions of TNF-α, IL-6, IL-1, INF-γ, intracellular nitric oxide synthase (iNOS), cyclooxygenase-2 (COX-2), and other cytokines by suppressing NF-κB in LPS-stimulated macrophage cells [57,58]. PHLTM also attenuated colitis by suppressing the synthesis of inflammatory cytokines and immune cell infiltration [59]. It also reduced the serum and alveolar neutrophil infiltration rate, downregulated TNF-α, IL-6, ICAM, and chemoattractant protein-1 (MCP-1), and inhibited NF-Kβ in animal models of lung injury [60,61]. However, the researchers in [60] also showed that this anti-inflammatory and NF-κβ inhibitory potential of PHLTM is associated with the overexpression of Nrf2 and HO-1, thus having an additional antioxidant effect. However, given the negative crosstalk between Nrf2 and NF-κB, these findings may also indicate the opposite and may suggest that PHLTM may exert its antioxidant potential by suppressing NF-κB. Additionally, other upstream mechanisms could be responsible for the anti-inflammatory effect of PHLTM. In support of this, the suppression of major mitogen-activated protein kinase members such as JNK, ERK1/2, and p38 was reported to be an upstream mechanism that mediates the inhibitory effect of several polyphenols on NF-κβ in LPS-stimulated macrophages [62,63].

Nevertheless, accumulating data suggest that the renal oxidative stress induced by hyperglycemia-derived ROS and the overwhelming or reduced expression of antioxidant enzymes are major upstream mechanisms that initiate and advance DN by promoting inflammation, fibrosis, and apoptosis [2,7,8,12]. In addition, hyperglycemia triggers an abnormal decline in the activity of Nrf2, which is normally activated by oxidative stress to alleviate the other pathological mechanisms listed above [20]. Indeed, the activation of Nrf2 not only promotes the upregulation of the cellular antioxidants but can also inhibit the activation of NF-κB and upregulate the expression of the anti-apoptotic protein Bcl2 [13,64]. Yet, the mechanisms by which hyperglycemia suppresses tissue Nrf2 signaling are still unclear. However, some of these mechanisms have been revealed during the last few years. Within this context, hyperglycemia was shown to promote the methylation of the ARE4 region, thus inhibiting the binding of Nrf2 to this region [65]. Also, hyperglycemia promotes the epigenetic modification-induced upregulation of keap1, which breaks down Nrf2 in the cytoplasm [66]. In addition, hyperglycemia upregulates the stress response protein regulated in the development of DNA damage 1 (REDD1), which, in turn, activates glycogen synthase kinase 3 (GSK3) to induce the phosphorylation-mediated degradation of Nrf2 [67]. Interestingly, the activation of Nrf2 has been suggested as a future therapy to treat DN by alleviating oxidative stress, inflammation, and apoptosis [11,68,69].

In this study, we also found that the nephroprotection afforded by PHLTM against STZ-mediated T1DM was mediated by antioxidant mechanisms that alleviated oxidative stress, reduced the generation of AGEs, and stimulated the nuclear activation of Nrf2 and the levels of GSH, HO-1, and SOD. These data suggest that the upregulation of Nrf2 is a central mechanism of protection underlying the action of PHLTM. In addition, the stimulatory effect of PHLTM on Nrf2 occurred at the transcription level, as it upregulated the mRNA and the protein levels of Nrf2 in the kidneys of both the control and diabetic rats. A similar stimulatory effect of PHLTM on the mRNA and protein levels was also observed previously in the livers of STZ-diabetic rats [27]. However, our results suggest that this activation of Nrf2 does not involve modulating the expression of keap1, since treatment with PHLTM did not affect the levels of keap1 in the control or diabetic kidneys. However, it could still be possible that PHLTM may interfere with the interaction between Nrf2 and keap1 to stimulate its nuclear translocation. This could be secondary to suppressing hyperglycemia, which normally inhibits Nrf2 by targeting the REDD1/GS3K pathway, or by promoting the methylation of the ARE4 region. In addition, given the negative crosstalk between NF-κβ and Nrf2, it could also be possible that the stimulatory effect of PHLTM on Nrf2 is secondary to its inhibitory effect on NF-κB, or vice versa. This was not examined here and will be considered in future studies to reveal the precise mechanism by which PHLTM activates Nrf2. As discussed before, the pharmacological antioxidant activity of PHLTM has been poorly examined. There are insufficient supporting studies in the literature to directly support our findings. However, extra support can be obtained by the previously reported antioxidant and Nrf2 stimulatory effect of phloretin. Indeed, phloretin inhibited H_2_O_2_-induced mitochondria-mediated apoptosis by reducing the generation of ROS and stimulating autophagy [70]. In addition, phloretin suppressed ROS generation and inhibited fibrosis in high-glucose-stimulated cardiac H9c2 cells by activating the Nrf2 antioxidant axis [71]. It also prevented oxidative damage in endothelial cells induced by palmitic acid by activating Nrf2 and upregulating GSH, SOD, and glutathione peroxidase (GPx) [72]. Furthermore, phloretin prevented rotenone-mediated brain neurodegeneration in a mouse model of Parkinson’s disease by activating Nrf2, upregulating antioxidants, and reducing oxidative stress [73]. In the same manner, phloretin alleviated hyperglycemia-mediated cardiomyopathy in H9c2 cells by stimulating the Nrf2/antioxidant axis and suppressing oxidative damage, hypertrophy, and fibrosis [71]. Moreover, phloretin also prevented renal and hepatic damage in streptozotocin (STZ)-diabetic rats [74].

In conclusion, the findings of this study are the first to show a potent nephroprotective effect of PHLTM in diabetic animals. The mechanisms of protection involve hypoglycemic, hypolipidemic, antioxidant, and anti-inflammatory effects. In addition, this protection is associated with activation of Nrf2 and suppression of NF-κβ. The findings of this presentation also encourage exploring the use of PHLTM in other nephropathies.

### Study Limitation

Despite these findings, this study still has some limitations. Even though we are reporting a renal protective effect of PHLTM in rats, further supporting evidence is needed to use this drug at the clinical level. For this reason, more pharmaceutical studies to determine the levels of PHLTM in the serum and urine for the used dose are highly recommended. In addition, this also should include characterization of PHLTM, including its structure, stability, plasma half-time, plasma levels/structure–activity relationships, and potential toxicity side effects. Furthermore, the mechanisms by which PHLTM activates Nrf2 and suppresses NF-κB should be examined in more advanced studies, such as those using knockout mice and targeting other regulatory pathways such as SIRT1, PKC, PI3K/Akt, etc.

## Figures and Tables

**Table 1:** Alterations in plasma and lipid profiles in all groups of rats.

		Control	Control + PHLTM	STZ-T1DM	STZ-T1DM + PHLTM
	Final body weight	534.1 ± 53.2	547.6 ± 49.3	322.2 ± 36.5 ^ab^	449.4 ± 39.5 ^abc^
Plasma	Glucose (mg/dL)	113.3 ± 10.3	92.5 ± 7.9 ^a^	344.2 ± 34.6 ^ab^	172.4 ± 25.4 ^abc^
	Insulin (ng/mL)	4.3 ± 0.6	4.1 ± 0.5	2.1 ± 0.2 ^ab^	1.9 ± 0.2 ^abc^
Serum	TGs (mg/dL)	83.4 ± 7.8	88.3 ± 8.2	169.3 ± 14.5 ^ab^	103.2 ± 15.9 ^abc^
	CHOL (mg/dL)	81.9 ± 8.4	88.5 ± 9.1	212.2 ± 18.7 ^ab^	148.2 ± 20.1 ^abc^
	LDL-c (mg/dL)	48.5 ± 5.7	44.3 ± 4.9	110.2 ± 9.5 ^ab^	76.3 ± 10.5 ^abc^
	HDL-c (mg/dL)	38.4 ± 3.6	40.8 ± 5.1	22.2 ± 2.7 ^ab^	31.2 ± 3.9 ^abc^
	FFAs (μmol/mg)	367.6 ± 35.9	293.3 ± 24.5 ^a^	663.3 ± 58.9 ^ab^	447.4 ± 48.1 ^abc^

Analysis was conducted using two-way ANOVA followed by Tukey’s test. Values are significantly different at *p* < 0.05. Data are expressed as means ± SD for *n* = 8 rats/group. ^a^ Significantly different from control (non-diabetic) rats (control). ^b^ Significantly different from control (non-diabetic) + PHLTM rats. ^c^ Significantly different from STZ-T1DM-treated rats.

**Table 2:** Kidney function tests in all experimental groups of the study.

	Control	Control + PHLTM	STZ-T1DM	STZ-T1DM + PHLTM
Serum				
Cr (μmol/L)	58.8 ± 6.9	54.4 ± 5.7	134.5 ± 11.6 ^ab^	67.5 ± 7.7 ^abc^
Albumin (g/dL)	3.89 ± 0.4	4.11 ± 0.6	1.63 ± 0.2 ^ab^	3.11 ± 0.4 ^abc^
Urine				
Volume (mL)	13.6 ± 1.9	12.7 ± 1.7	7.4 ± 0.9 ^ab^	12.2 ± 1.2 ^c^
Albumin (µg/dL)	31.6 ± 2.5	34.5 ± 2.1	356.2 ± 23.2 ^ab^	46.3 ± 4.8 ^abc^
Cr (µg/dL)	101.7 ± 8.5	108.1 ± 9.8	54.3 ± 4.9 ^ab^	90.3 ± 8.6 ^abc^
8-OHdG (ng/mL)	2.57 ± 0.4	2.73 ± 0.3	5.64 ± 0.6 ^ab^	3.22 ± 0.3 ^abc^
KIM-1 (pg/mL)	312.2 ± 27.5	324.4 ± 29.2	761.2 ± 66.5 ^ab^	415.9 ± 39.5 ^abc^
Nephrin pg/mL)	198.3 ± 20.4	212.4 ± 25.6	633.3 ± 54.6 ^ab^	274.4 ± 23.4 ^abc^
NGAL (pg/mL)	55.7 ± 6.7	51.2 ± 4.9	246.1 ± 22.8 ^ab^	88.9 ± 8.7 ^abc^

Analysis was conducted using two-way ANOVA followed by Tukey’s test. Values are significantly different at *p* < 0.05. Data are expressed as means ± SD for *n* = 8 rats/group. ^a^ Significantly different from control (non-diabetic) rats (control). ^b^ Significantly different from control (non-diabetic) + PHLTM rats. ^c^ Significantly different from STZ-T1DM-treated rats. Creatinine (Cr), kidney injury molecule-1 (KIM-1), 8-hydroxy-2′-deoxyguanosine (8-OHdG), neutrophil gelatinase-associated lipocalin (NGAL).

**Table 3:** Alteration in the renal markers of oxidative stress and inflammation in the kidneys of all experimental groups.

	Control	Control + PHLTM	STZ-T1DM	STZ-T1DM + PHLTM
MDA (nmol/g)	0.48 ± 0.05	0.51 ± 0.03 ^a^	1.66 ± 0.21 ^ab^	0.71 ± 0.06 ^abc^
GSH (μg/g)	46.7 ± 4.6	73.4 ± 5.8 ^a^	22.3 ± 3.1 ^ab^	48.5 ± 5.1 ^bc^
SOD (U/g)	14.6 ± 1.5	24.5 ± 2.1 ^a^	7.2 ± 0.8 ^ab^	15.2 ± 1.7 ^bc^
HO-1 (pg/g)	22.5 ± 2.5	36.8 ± 4.2 ^a^	10.9 ± 1.3 ^ab^	25.4 ± 2.1 ^bc^
AGEs (ng/g)	17.6 ± 1.1	15.4 ± 1.2	66.7 ± 4.9 ^ab^	27.4 ± 2.4 ^abc^
TNF-α (pg/g)	3.6 ± 0.42	4.2 ± 0.58	22.6 ± 1.9 ^ab^	8.8 ± 0.74 ^abc^
IL-6 (pg/g)	14.9 ± 1.3	16.4 ± 1.5	64.6 ± 3.7 ^ab^	22.6 ± 2.1 ^abc^

Analysis was conducted using two-way ANOVA followed by Tukey’s test. Values are significantly different at *p* < 0.05. Data are expressed as means ± SD for *n* = 8 rats/group. ^a^ Significantly different from control (non-diabetic) rats (control). ^b^ Significantly different from control (non-diabetic) + PHLTM rats. ^c^ Significantly different from STZ-T1DM-treated rats. Malondialdehyde (MDA), glutathione (GSH), superoxide dismutase (SOD), hemoxygenase-1 (HO-1), advanced glycation end products (AGEs), tumor necrosis factor α (TNF-α), interleukin 6 (IL-6).

**Table 4:** Alterations in some renal markers of apoptosis in all experimental groups.

	Control	Control + PHLTM	STZ-T1DM	STZ-T1DM + PHLTM
Bax (pg/g)	8.3 ± 0.7	9.1 ± 0.7	33.5 ± 2.7 ^ab^	13.2 ± 1.1 ^abc^
Bcl2 (nmol/g)	14.5 ± 1.2	22.9 ± 1.9 ^a^	6.5 ± 0.5 ^ab^	12.4 ± 0.9 ^bc^
Caspapse-3 (nmol/g)	4.8 ± 0.5	5.1 ± 0.5	22.6 ± 1.8 ^ab^	7.6 ± 0.8 ^c^
Bax/Bcl2 (%)	0.58 ± 0.06	0.38 ± 0.05 ^a^	4.9 ± 0. 7 ^ab^	1.1 ± 0.1 ^abc^

Analysis was conducted using two-way ANOVA followed by Tukey’s test. Values are significantly different at *p* < 0.05. Data are expressed as means ± SD for *n* = 8 rats/group. ^a^ Significantly different from control (non-diabetic) rats (control). ^b^ Significantly different from control (non-diabetic) + PHLTM rats. ^c^ Significantly different from STZ-T1DM-treated rats. Bax (BCL2-associated X), Bcl2 (B-cell lymphoma 2).

## Data Availability

The datasets used and analyzed during the current study are available from the corresponding author upon reasonable request.
